# Functional role of AKT signaling in bovine early embryonic development: potential link to embryotrophic actions of follistatin

**DOI:** 10.1186/s12958-017-0318-6

**Published:** 2018-01-08

**Authors:** Mohamed Ashry, Sandeep K. Rajput, Joseph K. Folger, Jason G. Knott, Nabil A. Hemeida, Omaima M. Kandil, Refaat S. Ragab, George W. Smith

**Affiliations:** 1 Laboratory of Mammalian Reproductive Biology and Genomics, Michigan State University, East Lansing, MI 48824 USA; 2 Developmental Epigenetics Laboratory, Michigan State University, East Lansing, MI 48824 USA; 3 Department of Animal Science, Michigan State University, East Lansing, MI 48824 USA; 4 Department of Theriogenology, Faculty of Veterinary Medicine, Cairo University, Giza, Egypt; 5 Department of Animal Reproduction and Artificial Insemination, Veterinary Research Division, National Research Center, Giza, Egypt

**Keywords:** AKT, Follistatin, TGF-β, AKT inhibitor, Bovine, Embryos

## Abstract

**Background:**

TGF-β signaling pathways regulate several crucial processes in female reproduction. AKT is a non-SMAD signaling pathway regulated by TGF-β ligands essential for oocyte maturation and early embryonic development in the mouse, but its regulatory role in bovine early embryonic development is not well established. Previously, we demonstrated a stimulatory role for follistatin (a binding protein for specific members of TGF-β superfamily) in early bovine embryonic development. The objectives of the present studies were to determine the functional role of AKT signaling in bovine early embryonic development and embryotrophic actions of follistatin.

**Methods:**

We used AKT inhibitors III and IV as pharmacological inhibitors of AKT signaling pathway during the first 72 h of in vitro embryo culture. Effects of AKT inhibition on early embryonic development and AKT phosphorylation were investigated in the presence or absence of exogenous follistatin.

**Results:**

Pharmacological inhibition of AKT signaling resulted in a significant reduction in early embryo cleavage, and development to the 8- to 16-cell and blastocyst stages (d7). Treatment with exogenous follistatin increased AKT phosphorylation and rescued the inhibitory effect of AKT inhibitors III and IV on AKT phosphorylation and early embryonic development.

**Conclusions:**

Collectively, results suggest a potential requirement of AKT for bovine early embryonic development, and suggest a potential role for follistatin in regulation of AKT signaling in early bovine embryos.

**Electronic supplementary material:**

The online version of this article (10.1186/s12958-017-0318-6) contains supplementary material, which is available to authorized users.

## Background

Poor oocyte quality limits the efficiency of in vitro embryo production in cattle and women [1]. Our previous studies demonstrated a positive association between the transcript abundance of follistatin and oocyte developmental competence using the prepubertal calf and brilliant cresyl blue screening models [2, 3]. We also found that maternally derived follistatin is essential for bovine early embryonic development and exogenous supplementation of follistatin during first 72 h (h) of in vitro embryo culture has a stimulatory effect on early cleavage, embryonic development to 8- to 16-cell and blastocyst stages and blastocyst cell lineage allocation [4]. The functional requirement of follistatin in oocyte and early embryonic development has been described in bovine and other mono-ovulatory species [5].

Follistatin is a transforming growth factor β (TGF-β) superfamily binding protein that may exert its embryotrophic effects through modulation of one or more of the SMAD [6, 7] or non-SMAD [8–10] signaling pathways. We previously demonstrated that the embryotrophic actions of follistatin on development to 8- to 16-cell and blastocyst stages are linked to the SMAD signaling pathways [11, 12]. However, how follistatin promotes early embryonic development, particularly early cleavage, is still not fully understood.

Follistatin was first identified as a high affinity Activin binding protein [13] and its binding affinity to select bone morphogenetic proteins (BMPs) has also been established [14, 15]. Both BMPs [16] and Activin [17] are major regulators of AKT signaling pathway that promotes cell cycle progression during G2/M transition. Studies have shown that AKT stimulates the transition from metaphase I (MI) to metaphase II (MII) in bovine [18], porcine [19], mouse [20, 21] and Xenopus [22] oocytes by activation of Phosphodiesterase 3A (PDE3A) and cyclin dependent kinase 1 (CDK1) required for resumption and regulation of meiosis. AKT was primarily identified as a serine/threonine specific protein kinase that functions downstream of Phosphatidylinositol 3-kinase (PI3K) [23]. Inhibition of AKT with a synthetic small molecule inhibitor (AKT inhibitor III) resulted in arrest of bovine oocytes at MI stage [18]. AKT plays an important role during entry of one cell mouse embryos into the first mitosis as inhibition of AKT resulted in arrest of cell cycle in G1 and G2 phases [24]. Moreover, inhibition of AKT activity compromised the development of mouse embryos to the blastocyst stage [25, 26]. Considering the role of AKT in cell cycle progression in oocytes and embryos, we hypothesized that AKT signaling is required for bovine early embryonic development and is linked to the embryotrophic actions of follistatin during in vitro embryo culture. To test this hypothesis, we determined the functional role of AKT in bovine early embryonic development and investigated the relationship between AKT signaling and the embryotrophic actions of follistatin. Effects of treatment with the AKT inhibitors III and IV on development of early bovine embryos and AKT phosphorylation were analyzed. Effects of exogenous follistatin supplementation on AKT phosphorylation and developmental potential of AKT inhibitor treated embryos were also investigated.

## Methods

All chemicals and reagents used were purchased from Sigma- Aldrich (St. Louis, MO) unless stated otherwise.

### Oocyte collection and in vitro embryo production

Bovine oocytes were retrieved from ovaries collected at local abattoir in the state of Michigan. Oocyte aspiration, in vitro maturation (IVM), in vitro fertilization (IVF), and in vitro embryo culture (IVC) were performed as previously described [3, 27]. Briefly, morphologically good quality cumulus oocyte complexes (COCs), which have three or more layers of compact cumulus cell and granular, homogenous cytoplasm, were matured in TCM-199 media [supplemented with 50 μg/ml gentamycin sulfate, 0.2 mM sodium pyruvate, 3.67 nM 17β-estradiol, 15.6 nM bovine FSH (Sioux Biochemical, Sioux Center, IA), 156 nM bovine LH (Sioux Biochemical), and 10% *v*/v defined fetal bovine serum (FBS) (Hyclone, Logan, UT)] at 38.5 °C in 5% CO_2_ and humidified air for 24 h. IVF was conducted using motile spermatozoa separated by Percoll gradient technique from frozen thawed bovine semen. The matured COCs were co-incubated with motile spermatozoa (1 × 10^6^ sperm/ml) for 18–20 h in fertilization media FIV (114 mM NaCl, 25 mM NaHCO_3_, 3.2 mM KCl, 0.34 mM NaH_2_PO_4_, 0.183 mM penicillin-G, 16.6 mM sodium lactate, 0.5 mM MgCl_2_6H_2_O, 2.7 mM CaCl_2_2H_2_O, 0.2 mM sodium pyruvate, 6 mg/ml BSA, and 1.5 U of heparin) at 38.5 °C in 5% CO_2_ and humidified air. After IVM and IVF, the cumulus cells were stripped off by vortexing for 6 min. Then, presumptive zygotes were washed and cultured in potassium simplex optimization medium (KSOM; EMD Millipore, Billerica, MA) supplemented with 0.3% fatty acid free bovine serum albumin (BSA) until 72 h post insemination (hpi). Then, 8- to 16-cell stage embryos were separated and cultured in fresh KSOM medium supplemented with 0.3% BSA and 10% FBS under same conditions until d 7.

### Effect of AKT inhibitors III & IV on early embryonic development

**Fig. 1:**
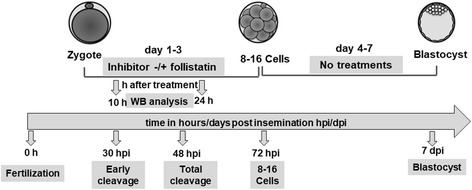
Experimental design. To establish the role of AKT in early bovine embryonic development, bovine zygotes were cultured in the presence or absence of different doses of AKT inhibitors III or IV for 3 days. The optimized dose of each inhibitor was supplemented with or without 10 ng/ml follistatin to determine the effects on AKT phosphorylation at 10 h after treatment and development of AKT inhibitor treated embryos. On day 3 of development, embryos were washed and cultured in fresh media without any treatment for an additional 4 days. Indices of embryo development including early cleavage rate (30 hpi), total cleavage rate (48 hpi), percentage of embryos developing to the 8- to 16-cell stage (72 hpi), and percentage of embryos developing to the blastocyst stage (day 7) were recorded

AKT inhibitor III, known as SH6, is a cell permeable reversible substrate competitive phosphatidylinositol analog that prevents the generation of PIP3 by PIK3 which is required for AKT phosphorylation. AKT inhibitor IV is a cell permeable reversible benzimidazole compound that inhibits AKT phosphorylation/activation by targeting the ATP binding site of a kinase upstream of AKT, but downstream of PI3K [28]. The effects of AKT inhibitors III and IV treatment on early embryonic development were investigated as described in Fig. 1. Presumptive zygotes were cultured in KSOM medium supplemented with 0.3% BSA in the presence of 0, 25, 50 or 75 μM AKT inhibitor III (ALX-270-350 - Enzo Life Sciences, Farmingdale, NY), or 0, 1.5, 2.5 or 3.5 μM AKT inhibitor IV (Sigma, 124,011-1MG) until 72 hpi (*n* = 25–30 presumptive zygotes/group, *n* = 4 replicates/inhibitor). Embryos at 8- to 16-cell stage were separated, washed and cultured in fresh KSOM medium supplemented with 0.3% BSA and 10% FBS in the absence of inhibitor until d 7. The effects of AKT inhibition on indices of embryo developmental progression including early cleavage rate determined at 30 hpi, total cleavage rate determined at 48 hpi, percentage of embryos developing to the 8- to 16-cell stage at 72 hpi and percentage of embryos developing to the blastocyst stage at d 7 were recorded.

### Effect of AKT inhibitors III & IV on AKT phosphorylation in early bovine embryos

To investigate the effect of AKT inhibitors III & IV treatment on AKT signaling activity in bovine embryo, AKT-Ser473 and AKT-Thr308 phosphorylation levels were analyzed. Presumptive zygotes were cultured in KSOM medium supplemented with 0.3% BSA in the presence of 0, 25, 50 or 75 μM AKT inhibitor III (Fig. 1) [18], or 0, 1.5, 2.5 or 3.5 μM AKT inhibitor IV for 10 h. Then, samples were collected for Western blot analysis of phosphorylated (p)-AKT-Ser473 or pAKT-Thr308, total (t)-AKT and actin (*n* = 3 replicates/inhibitor/phosphorylation site, *n* = 20 presumptive zygotes/group). All the doses of AKT inhibitors used in this study were selected based on previously published studies in bovine and mice [18, 19, 21].

### Effects of follistatin supplementation on the developmental capacity of AKT inhibitor treated bovine embryos

Experiment was performed as described in Fig. 1. After IVM and IVF, presumptive zygotes were cultured with 0 or 10 ng/ml recombinant human follistatin (R&D Systems, Minneapolis, MN) [4] in the presence or absence of AKT inhibitor III (75 μM) or AKT inhibitor IV (3.5 μM) until 72 hpi (*n* = 4 replicates/inhibitor, *n* = 20–30 zygotes/group). Then, 8- to 16-cell stage embryos from each group were separated and cultured in fresh KSOM medium supplemented with 0.3% BSA and 10% FBS without any treatment until d 7. Effects of exogenous follistatin on early and total cleavage, development to 8–16-cell and d7 blastocyst rates were recorded.

### Effect of follistatin supplementation on activity of AKT signaling pathway in AKT inhibitor treated early bovine embryos

Effects of follistatin supplementation on AKT signaling activity were investigated by analysis of AKT phosphorylation levels at 10 and 24 h after follistatin supplementation in the presence and absence of AKT inhibitor III or IV (Fig. 1). Presumptive zygotes were cultured with 0 or 10 ng/ml recombinant human follistatin in the presence or absence of AKT inhibitor III (75 μM) or AKT inhibitor IV (3.5 μM) during the initial 72 h of in vitro embryo culture. Samples were collected at 10 h after treatment administration (*n* = 6 replicates/inhibitor, *n* = 20 zygotes/group). For the second experiment, follistatin (0 or 10 ng/ml) was supplemented during the same time window and samples collected at 24 h after treatment administration (*n* = 5 replicates/inhibitor, *n* = 20 zygotes/group). Samples were subjected to Western blot analysis of pAKT-Ser473, pAKT-Thr308, tAKT and actin.

### Western blot analysis

Western blot analysis was performed as described before [3]. Proteins (20 oocytes/embryos per sample) were separated by SDS-PAGE electrophoresis using 4–20% gradient pre-casted gel (Bio-Rad, Hercules, CA) and transferred to a polyvinylidene difluoride (PVDF, Millipore) membrane. After transfer, membranes were blocked with 5% BSA in Tris Buffered Saline with 0.5% Tween 20 (TBST) for 1 h at room temperature before overnight incubation with the appropriate primary antibody diluted in TBST with 5% BSA at 4 °C. The membrane was then washed and incubated with the appropriate HRP-conjugated secondary antibody diluted in TBST with 5% BSA for 1 h at room temperature. Protein signals were detected with SuperSignal West Dura Chemiluminescent Substrate (Thermo Scientific, Waltham, MA) and Images were captured using myECL Imager (Thermo Scientific). ImageJ software was used to measure the band intensities of the target proteins [29]. Relative abundance of each protein was normalized to the total–actin expression in corresponding lane, and phosphorylation level was expressed as phosphorylated (p) AKT/total (t) AKT. Membranes were probed sequentially with primary pAKT-Ser473 rabbit polyclonal antibody [1:1000 (Vol/Vol), Santa Cruz, sc-7985-R] or pAKT-Thr308 rabbit polyclonal antibody [1:1000 (Vol/Vol), Cell Signaling Technology, 9275]. After detection of pAKT membranes were striped with restore Western blot stripping buffer (Thermo Scientific) for 15 min at room temperature and re-probed with tAKT Rabbit polyclonal antibody [1:2000 (Vol/Vol), Santa Cruz, sc-8312]. After detection of tAKT, membranes were stripped and re-probed with actin monoclonal antibody (1:5000 (Vol/Vol) Millipore; MAB1501). HRP-conjugated Anti-Rabbit-IgG (Cell Signaling Technology, 7074), and Anti-Mouse-IgG (Thermo Scientific, A16011) were used as secondary antibodies at a 1:5000 (Vol/Vol) dilution.

### Statistical analysis

Differences in phosphorylation levels obtained by Western-blot analysis were analyzed by ANOVA using the general linear models’ procedure of SAS (SAS Institute Inc., Cary, NC). For experiments studying the effects of AKT inhibitors and follistatin supplementation on early embryonic development, data were arcsine transformed prior to analysis by mixed linear models’ analysis procedures. Differences among treatment means were compared using Fisher’s protected least significant difference test. In all cases data are presented as untransformed mean ± standard error (*P* < 0.05).

## Results

### AKT inhibition during the initial 72 h of in vitro culture reduced indices of embryonic developmental progression

**Fig. 2:**
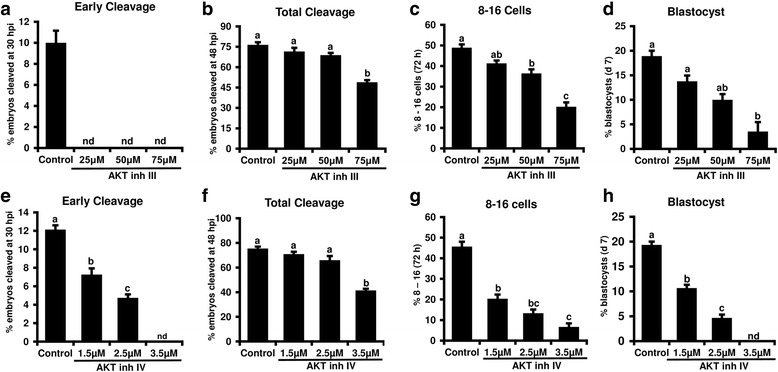
Effects of AKT inhibitors III and IV treatment on bovine early embryonic development. Presumptive zygotes were cultured in the presence of 0, 25, 50 or 75 μM AKT inhibitor III or 0, 1.5, 2.5 or 3.5 μM AKT inhibitor IV until 72 hpi, then washed and cultured in fresh culture media lacking inhibitors until d 7 (*n* = 4 replicates/inhibitor, *n* = 25–30 embryos/treatment). Effects of each AKT inhibitor treatment on (**a**, **e**) early cleavage, (**b**, **f**) total cleavage, (**c**, **g**) development to 8- to 16-cell stage and (**d**, **h**) d7 blastocyst rates were determined. Data are expressed as mean ± SEM. Values with different superscripts among treatments indicate significant differences (*P* < 0.05)

To elucidate the potential role of AKT in bovine early embryogenesis, effects of AKT inhibitor III and AKT inhibitor IV supplementation (during the initial 72 h of in vitro embryo culture) on different developmental indices (early cleavage, total cleavage, 8- to 16-cell and blastocyst formation rates) were determined. The addition of 25, 50 and 75 μM AKT inhibitor III to the culture medium delayed early cleavage therefore no cleavage was observed at 30 hpi (Fig. 2a). Treatment with AKT inhibitor III significantly reduced the total cleavage rate, percentage of embryos that reached 8- to 16-cell stage at 72 hpi, and d 7 blastocyst rate relative to controls at 75 μM dose (Fig. 2b-d). However, no significant effects on total cleavage were observed in response to both 25 and 50 μM doses compared with control (Fig. 2b, c). Additionally, the 25-mM dose didn’t impact the development to 8-to 16-cell or blastocyst stages (Fig. 2c-d). The addition of AKT inhibitor IV resulted in a dose dependent reduction in early cleavage, development to 8- to 16-cell and blastocyst stage with maximum response observed for 3.5 μM dose which delayed early cleavage and completely blocked development to blastocyst. Total cleavage wasn’t impacted by lower doses of AKT inhibitor IV (1.5, 2.5 μM) whereas the higher dose (3.5 μM) significantly reduced the number of embryos reaching two cells at 48 hpi (Fig. 2e-h). Results demonstrate that inhibition of AKT delayed early embryonic cleavage at 30 hpi, and consequently reduced development to the later endpoints, suggesting a potential requirement of AKT for early embryonic development.

### Effects of AKT inhibitors III & IV on AKT phosphorylation in early bovine embryos

**Fig. 3:**
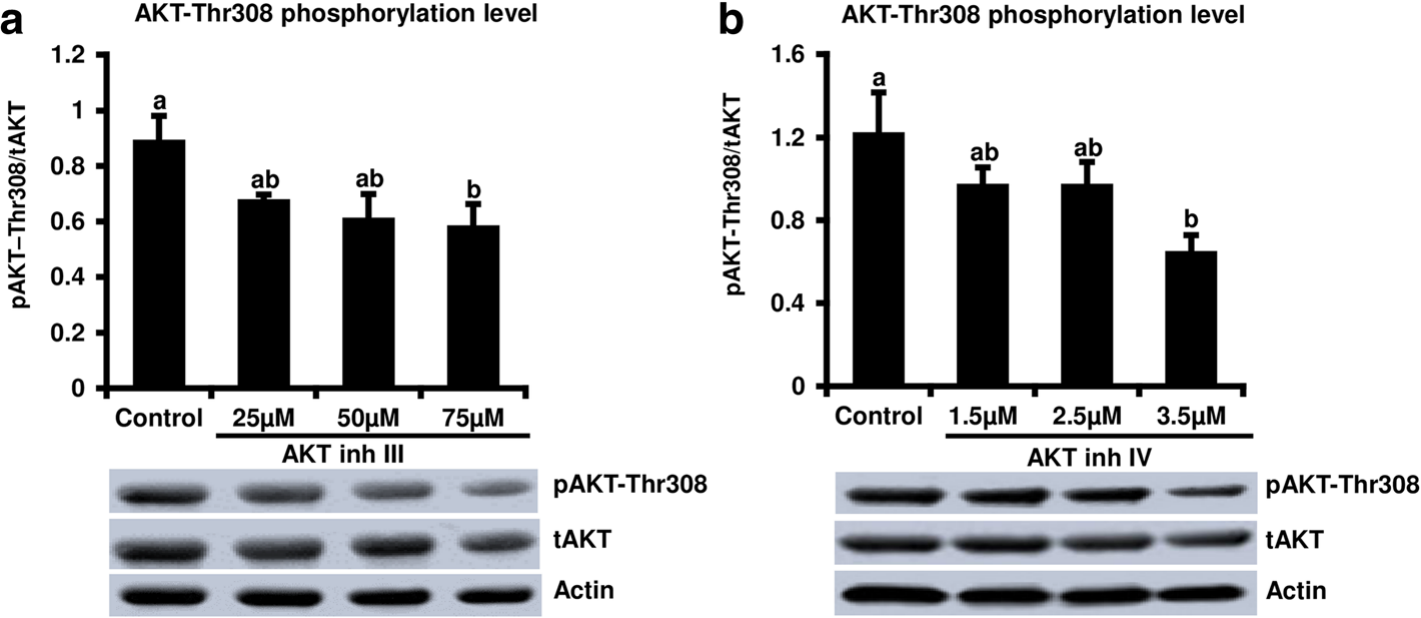
Effect of AKT inhibitors III and IV treatments on AKT-Thr308 phosphorylation levels in early bovine embryos*.* Presumptive zygotes were cultured in presence of 0, 25, 50 or 75 μM AKT inhibitor III or 0, 1.5, 2.5 or 3.5 μM AKT inhibitor IV for 10 h, then subjected to Western blot for phosphorylated (p)AKt-Th308, total AKT (tAKT) and actin analysis (*n* = 3 replicates, *n* = 20 embryos/treatment). Data were normalized relative to abundance of actin and phosphorylation levels were expressed as pAKT/tAKT (**a**, **b**). Representative Western blot images are shown. Data are expressed as mean ± SEM. Values with different superscripts among treatments indicate significant differences (*P* < 0.05)

Dose response studies were conducted to determine the effects of different concentrations of AKT inhibitor III (25, 50, and 75 μM) or AKT inhibitor IV (1.5, 2.5 and 3.5 μM) on AKT-Ser 473 and Thr308 phosphorylation levels in early embryos after 10 h of treatment. AKT-Thr308 phosphorylation level was significantly reduced in response to high doses of AKT inhibitor III (75 μM) and AKT inhibitor IV (3.5 μM) whereas slight non-significant reduction (*P* < .09) in phosphorylation level was also observed with lower doses of AKT inhibitor III (25 or 50 μM) or AKT inhibitor IV (1.5 and 2.5 μM) (Fig. 3a, b). On the other hand, AKT-Ser473 phosphorylation level was not impacted by treatment with the 25 μM AKT inhibitor III dose, whereas 50, and 75 μM doses, resulted in a significant reduction after 10 h of supplementation (Additional file 1: Figure S1a). Treatment with AKT inhibitor IV didn’t show any effect on AKT-Ser437 phosphorylation at this time point (Additional file 1: Figure S1b).

### Follistatin supplementation rescues the adverse effects of AKT inhibition on early embryo development

**Fig. 4:**
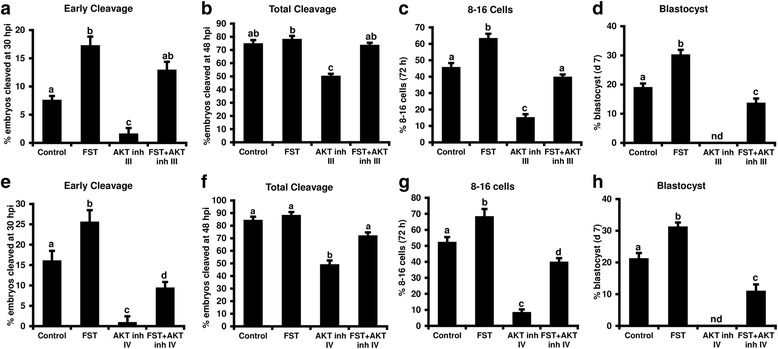
Effect of follistatin supplementation on development of AKT inhibitors treated bovine embryos. Presumptive zygotes were cultured with 0 or 10 ng/ml recombinant human follistatin in the presence or absence of AKT inhibitor III (75 μM) or AKT inhibitor IV (3.5 μM) until 72 hpi then washed and cultured in fresh media lacking inhibitors and follistatin until d 7 (*n* = 4 replicates/inhibitor, *n* = 25–30 embryos/treatment). Effects of follistatin on multiple developmental endpoints for AKT inhibitor III or AKT inhibitor IV treated embryos were determined including; (**a**, **e**) early cleavage, (**b**, **f**) total cleavage, (**c**, **g**) development to 8- to 16-cell stage and (**d**, **h**) d7 blastocyst rates. Data are expressed as mean ± SEM. Values with different superscripts among treatments indicate significant differences (*P* < 0.05)

Our previous studies established a functional requirement of maternally derived follistatin for bovine early embryogenesis and embryotrophic effects of exogenous follistatin supplementation on early embryo development including enhanced early cleavage, and increased blastocyst formation rate and trophectoderm cell numbers. The AKT signaling pathway is regulated by members of TFG-β superfamily [8]. Hence, we investigated if follistatin supplementation can rescue the negative effects of AKT inhibition on early embryonic development. In the absence of the AKT inhibitors, follistatin supplementation (10 ng/ml) significantly increased the proportion of embryos reaching 2-cell stage at 30 hpi (early cleavage), the proportion of embryos reaching 8- to 16-cell stage at 72 h and d7 blastocyst rates compared with untreated controls. In addition, follistatin supplementation rescued the inhibitory effects of AKT inhibitors on early embryonic development (Fig. 4a-h). Follistatin supplementation was able to rescue the effects of AKT inhibitor III on early cleavage, total cleavage and development to 8- to 16-cell stage to levels similar to controls (Fig. 4a-c), and to partially rescue the effects of AKT inhibitor III on blastocyst development rate (Fig. 4d). Using AKT inhibitor IV with same experimental design, we observed that follistatin supplementation (10 ng/ml) partially rescued the negative effects of AKT inhibitor IV on total cleavage, early cleavage, 8- to 16-cell and blastocysts development rates (Fig. 4e-h). Collectively, results suggest a potential requirement of AKT for bovine early embryonic development, and a possible relationship between the embryotrophic actions of follistatin and the AKT signaling pathway.

### Follistatin supplementation modulates the AKT signaling pathway in early bovine embryos

**Fig. 5:**
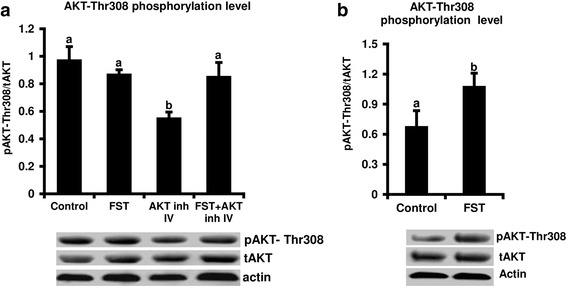
Effect of follistatin treatment on AKT-Thr308 phosphorylation levels in early bovine embryos. Presumptive zygotes were cultured with 0 or 10 ng/ml recombinant human follistatin in the presence or absence of AKT inhibitor IV (3.5 μM) for 10 h (*n* = 5 replicates, *n* = 20 zygotes/group) (**a**), or Presumptive zygotes were cultured in the presence or absence of 10 ng/ml follistatin for 24 h (*n* = 5 replicates, *n* = 20 zygotes/group) (**b**). Samples were subjected to Western blot analysis for pAKT-Thr308, tAKT and Actin. Expression levels were normalized to the abundance of an endogenous control actin. Phosphorylation level was expressed as pAKT/tAKT. Data are expressed as mean ± standard error. Values with different superscripts among treatments indicate significant differences (*P* < 0.05). Representative Western blots are shown

Our results revealed that exogenous follistatin could rescue the negative effects of AKT inhibition on various developmental endpoints in bovine embryos. Therefore, we analyzed the effect of exogenous follistatin supplementation on AKT signaling activity in the presence or absence of AKT inhibitors to determine whether follistatin rescue the effects of AKT inhibition through modulation of AKT signaling. Western blot analysis showed that AKT inhibitor IV treatment resulted in a significant reduction in AKT-Thr308 phosphorylation level in zygotes 10 h post treatment that was rescued by supplementation with exogenous follistatin. However, no effect of follistatin treatment on basal levels of AKT phosphorylation was observed in the absence of inhibitor treatment (Fig. 5a). Similar pattern was observed in response to AKT inhibitor III treatment (Additional file 2: Figure S2a). We further investigated if follistatin supplementation has any effect on basal AKT phosphorylation at a later time point. At 24 h post treatment administration, follistatin supplementation resulted in a significant increase in AKT phosphorylation relative to embryos cultured in the absence of follistatin (Fig. 5b, Additional file 2: Figure S2b).

## Discussion

A growing body of evidence supports an important intrinsic role for follistatin in bovine oocyte quality and early embryo developmental progression in vitro [3, 4, 11, 12]. While follistatin is best known for its ability to bind and inhibit activity of select TGF-β superfamily ligands, the intrinsic ligands and signaling pathways linked to trophic actions of follistatin on early embryos are not known. Results of the present study demonstrated that pharmacological inhibition of AKT signaling pathway in early bovine embryos reduced their developmental capacity as assessed by early cleavage, total cleavage and rates of development to 8–16-cell and blastocyst stages. Follistatin supplementation during the first 72 h of in vitro embryo culture rescued the adverse effects of AKT signaling inhibition on embryo development and increased the level of AKT phosphorylation, suggesting a potential role for follistatin in regulation of AKT signaling in early bovine embryos. Results demonstrate follistatin regulation of non-canonical TGF-beta superfamily signaling in bovine embryos and a functional link to developmental progression.

AKT is serine/threonine-specific protein kinase that regulates crucial cellular processes such as glucose metabolism, transcription, cellular growth and proliferation [30–32]. Full activation of AKT requires translocation to the plasma membrane and phosphorylation at Thr308 and Ser473 motifs [33]. Previous studies have established a functional role of AKT during oocyte maturation in various species including bovine [18] and in early embryogenesis in mouse and pigs [19, 20, 34]. In bovine embryo, activation of PI3K/AKT pathway by insulin like growth factor − 1 (IGF-1) has been shown to block heat shock induced apoptosis [35]. In addition, pharmacological inhibition of PI3K, upstream kinase of AKT, using LY294002 resulted in significant decrease in total cleavage, 8 cells and blastocyst development rates in bovine [36]. In the present study, we used two different pharmacological inhibitors of AKT activity to investigate its role in early bovine embryo development. Pharmacological inhibition was selected over siRNA mediated knockdown because inhibitors are membrane permeable and can function immediately by specifically targeting AKT activity without affecting its total protein abundance in embryo. As inhibitors can function in reversible manner, this allowed us to inhibit AKT signaling for define period (d1–3) of in vitro embryo culture in this study. Our does dependent study showed that AKT inhibitor III at 75 μm or AKT inhibitor IV at 3.5 μM doses exhibited about 50% reduction in AKT phosphorylation levels and therefore were selected as most effective dose to inhibit AKT signaling. These results were in accordance with the previously published studies in bovine and mice [18, 19, 21]. In addition, higher doses of AKT inhibitor III used in this study has been previously reported to have no nonspecific effect on other kinases downstream to PDK1 [18].

Our results showed that inhibition of AKT during first 3 days of in vitro embryo culture (before zygotic genome activation) resulted in pronounced reduction in multiple indices of early embryonic development. Early embryonic cleavage at 30 hpi was not detected for all tested doses of AKT inhibitor III. Total cleavage, rates of development to 8- to 16-cell and day 7 blastocyst rates were significantly reduced in embryos treated with the highest dose of AKT inhibitor III. We observed similar effects of AKT signaling inhibition on embryo development using AKT inhibitor IV. Data from other species [37, 38] suggest that AKT is implicated in regulation of mitotic cell cycle transition from G2 to M phase through activation of M-phase promoting factor (MPF). Therefore, upon inhibition of AKT, the MPF complex remains inactive and the cells are arrested in G1 or G2 stages of the cell cycle [24, 39]. Reduction of AKT phosphorylation/activity may delay cell cycle progression [40] or compromise the developmental capacity of treated embryos. Therefore, early cleavage at 30 hpi was not detected, and total cleavage, development to 8- to 16-cell and blastocyst stages were reduced compared with untreated embryos. Collectively, these results demonstrated that inhibition of AKT signaling using two different AKT inhibitors similarly reduced the development of early bovine embryos in vitro suggesting an important functional role of AKT signaling in early bovine embryogenesis. However, further mechanistic studies are required to conclusively elucidate how the AKT signaling pathway regulates early embryonic development in bovine.

We previously established a positive association between follistatin and oocyte developmental competence [2, 3]. Supplementation of exogenous follistatin during in vitro embryo culture has embryotropic effects in bovine and non-human primates [4, 41]. Although, follistatin was originally identified as an activin antagonist [13], the embryotropic actions of follistatin are presumably not mediated by inhibition of activin action as exogenous supplementation of activin mimicked the embryotrophic effects of follistatin [4]. As a TGF-β superfamily growth factor binding protein, follistatin may exert its embryotrophic actions by modulating SMAD dependent (SMAD1/5/8 or SMAD2/3) or non*-*SMAD dependent (AKT, ERK, P38 and JNK) pathways implicated in signaling by this growth factor family [8]. Previous studies in our laboratory demonstrated that, exogenous follistatin supplementation rescued the effects of SMAD2/3 and SMAD4 inhibition/knockdown on early cleavage but not the effects on development to 8- to 16-cell and blastocyst stages, suggesting that the embryotrophic effects of follistatin on early cleavage are SMAD independent [11, 12]. In the current study, the effects of follistatin supplementation on development of AKT inhibitor treated embryos were investigated. Treatment with 10 ng/ml follistatin during the initial 72 h of in vitro embryo culture reversed the inhibitory effects of AKT inhibitor III on early cleavage, total cleavage and development to the 8- to 16-cell stage and partially rescued development to the blastocyst stage. Results also demonstrated that AKT inhibitors significantly reduced the levels of AKT phosphorylation in the absence of follistatin. In addition, follistatin treatment not only reverse the AKT inhibitors effect on phosphorylation level but also increase the AKT phosphorylation when supplemented independently for 24 h. These results suggest that follistatin may be playing a role in returning the AKT phosphorylation to the levels required for rescuing the adverse effect of AKT inhibitors on early cleavage. Collectively, results suggest a potential functional link between follistatin action and AKT signaling linked to its embryotropic actions in bovine embryos. As the effects of other signaling pathways can’t be disregarded, further studies may be required to elucidate the effects of inhibition of multiple signaling pathways on the development of early bovine embryos and to dissect the specific effects of each signaling pathway.

Effects of TGF-β superfamily stimulation on AKT signaling appear to be pleiotropic and cell type dependent. It has been demonstrated that members of TGF-β superfamily activate PI3K pathway through phosphorylation of its downstream effector AKT [42–44]. TGF-β can induce a physical interaction between the p85 regulatory subunit of PI3K and the TβRII and TβRI receptors converting phosphatidylinositol-4,5-bisphosphate (PIP_2_) to phosphatidylinositol-3,4,5-triphosphate (PIP_3_) and subsequently AKT phosphorylation [45–47]. Studies on somatic cell lines indicate that follistatin treatment activates PI3K/AKT signaling pathway [48, 49]. In the present studies, follistatin treatment reversed the inhibitory effects of AKT inhibitor treatment on AKT phosphorylation levels at 10 h post treatment, but a follistatin dependent increase in basal AKT phosphorylation was not observed in the absence of AKT inhibitor treatment. However, follistatin treatment did increase basal AKT phosphorylation levels 24 h post treatment administration. The exact reason for time dependent effects on AKT signaling observed in response to follistatin treatment are not known but may be reflecting time/treatment induced differences in endogenous growth factor milieu and bioaffinity for specific ligands. Results suggest that follistatin plays a role in regulation of AKT signaling in early bovine embryos. However, AKT seems to not be required for the embryotrophic actions of follistatin.

## Conclusions

Results of the present study demonstrate that inhibition of AKT signaling reduce the developmental competence of early bovine embryos suggesting a potential requirement of AKT signaling for bovine early embryonic development. Follistatin supplementation rescued the developmental competence of AKT inhibitor treated embryos suggesting that the examined embryotrophic effects of follistatin do not require AKT signaling. However, follistatin supplementation increased the relative abundance of pAKT in AKT inhibitor treated embryos and increased the level of AKT phosphorylation when supplemented without AKT inhibitor, suggesting that follistatin plays a role in regulation of AKT signaling in early bovine embryos. Together, the results reported here, provide additional insights into understanding the regulation of early embryonic development and the mechanism of action of the embryotrophic agent, follistatin, in early bovine embryos.

## Additional files

Additional file 1: Figure S1. Effect of AKT inhibitors III and IV treatments on AKT-Ser473 phosphorylation levels in early bovine embryos. IVF embryos were cultured in presence of 0, 25, 50 or 75 μM AKT inhibitor III or 0, 1.5, 2.5 or 3.5 μM AKT inhibitor IV for 10 h, then subjected to Western blot for pAKT-Ser473, tAKT and actin analysis (*n* = 3 replicates/antibody, *n* = 20 embryos/treatment). Data were normalized relative to abundance of actin and phosphorylation levels (**a, b**) were expressed as pAKT/tAKT. Representative Western blot images are shown. Data are expressed as mean ± SEM. Values with different superscripts among treatments indicate significant differences (*P* < 0.05). (TIFF 650 kb)

Additional file 2: Figure S2. Effect of follistatin treatment on AKT-Ser473 phosphorylation in early bovine embryos. Presumptive zygotes were cultured with 0 or 10 ng/ml recombinant human follistatin in the presence or absence of AKT inhibitor III (75 μM) for 10 h (*n* = 5 replicates, *n* = 20 zygotes/group) (**a**), or Presumptive zygotes were cultured in the presence or absence of 10 ng/ml follistatin for 24 h (*n* = 5 replicates, *n* = 20 zygotes/group) (**b**). Samples were subjected to Western blot analysis for pAKT-Ser473, tAKT and Actin. Expression levels were normalized to the abundance of an endogenous control actin. Phosphorylation level was expressed as pAKT/tAKT. Data are expressed as mean ± standard error. Values with different superscripts among treatments indicate significant differences (*P* < 0.05). Representative Western blots are shown. (TIFF 542 kb)

## Abbreviations

AKT/PKB: Protein Kinase B
BMPs: Bone Morphogenetic Proteins
BSA: Bovine Serum Albumin
CDK1: Cyclin Dependent Kinase 1
COCs: Cumulus Oocyte Complexes
D: Day
Dpi: Days post insemination
FBS: Fetal Bovine Serum
FSH: Follicle Stimulating Hormone
H: Hour
Hpi: Hour Post Insemination
IGF-1: Insulin Like Growth Factor − 1
Inh: Inhibitor
IVC: In Vitro Culture
IVF: In Vitro Fertilization
IVM: In Vitro Maturation
KSOM: Potassium Simplex Optimization Medium
LH: Luteinizing Hormone
MI: Metaphase I
MII: Metaphase II
pAKT: Phosphorylated AKT
PDE3A: Phosphodiesterase 3A
PVDF: Polyvinylidene Difluoride
Ser: Serine
tAKT: Total AKT
TBST: Tris Buffered Saline
TGF-β: Transforming growth factor β
Thr: Threonine
WB: Western blotting
μM: Micro molar
