# Supplementary figures and images for: Functional role of AKT signaling in bovine early embryonic development: potential link to embryotrophic actions of follistatin
Source: Reprod Biol Endocrinol. 2018 Jan 8;16:1. doi: 10.1186/s12958-017-0318-6 (PMC5759257; doi:10.1186/s12958-017-0318-6)

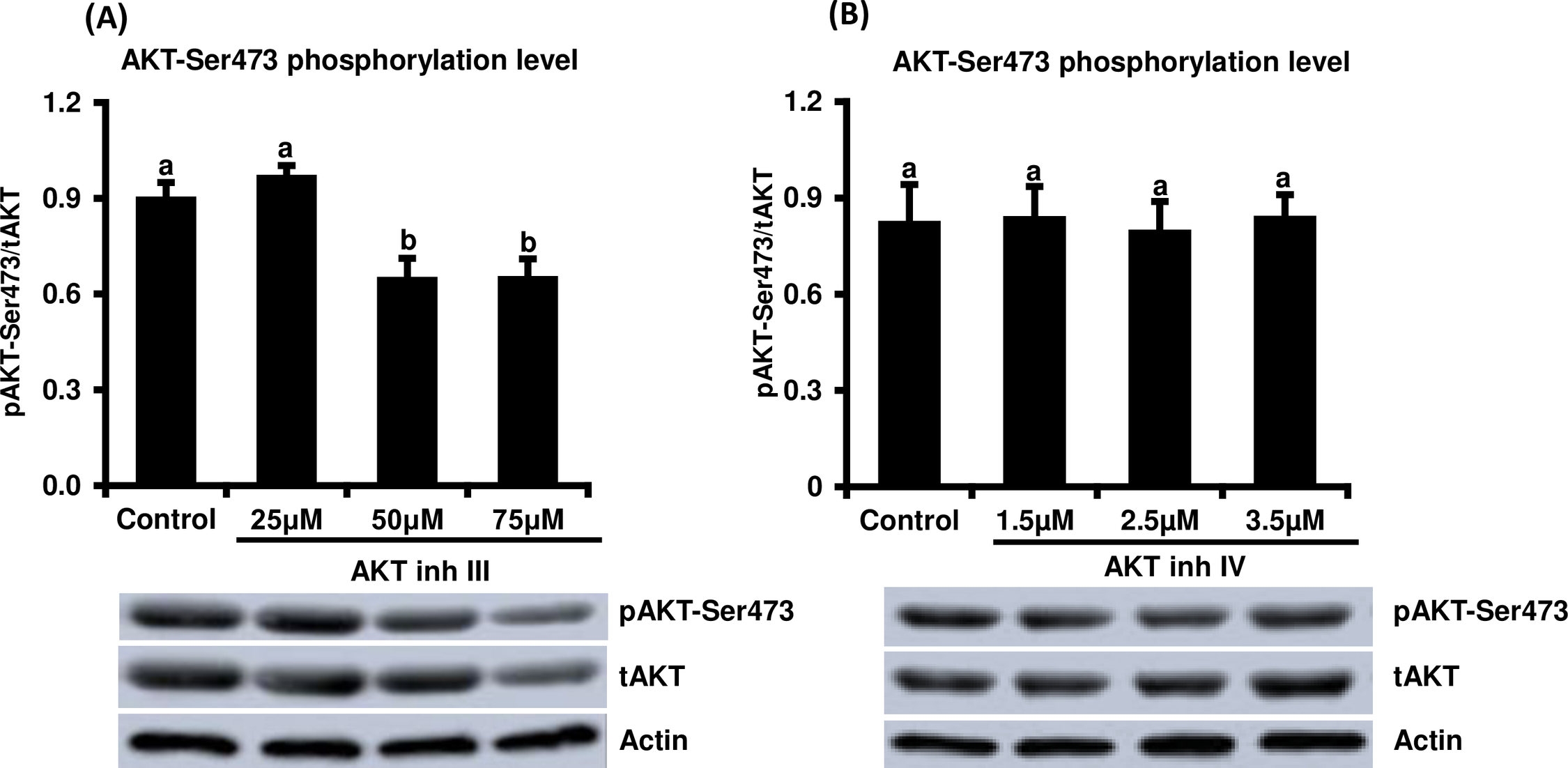

Supplement: Supplementary file 1 — Effect of AKT inhibitors III and IV treatments on AKT-Ser473 phosphorylation levels in early bovine embryos. IVF embryos were cultured in presence of 0, 25, 50 or 75 μM AKT inhibitor III or 0, 1.5, 2.5 or 3.5 μM AKT inhibitor IV for 10 h, then subjected to Western blot for pAKT-Ser473, tAKT and actin analysis (n = 3 replicates/antibody, n = 20 embryos/treatment). Data were normalized relative to abundance of actin and phosphorylation levels (a, b) were expressed as pAKT/tAKT. Representative Western blot images are shown. Data are expressed as mean ± SEM. Values with different superscripts among treatments indicate significant differences (P < 0.05). (TIFF 650 kb) [file 12958_2017_318_MOESM1_ESM.tif]

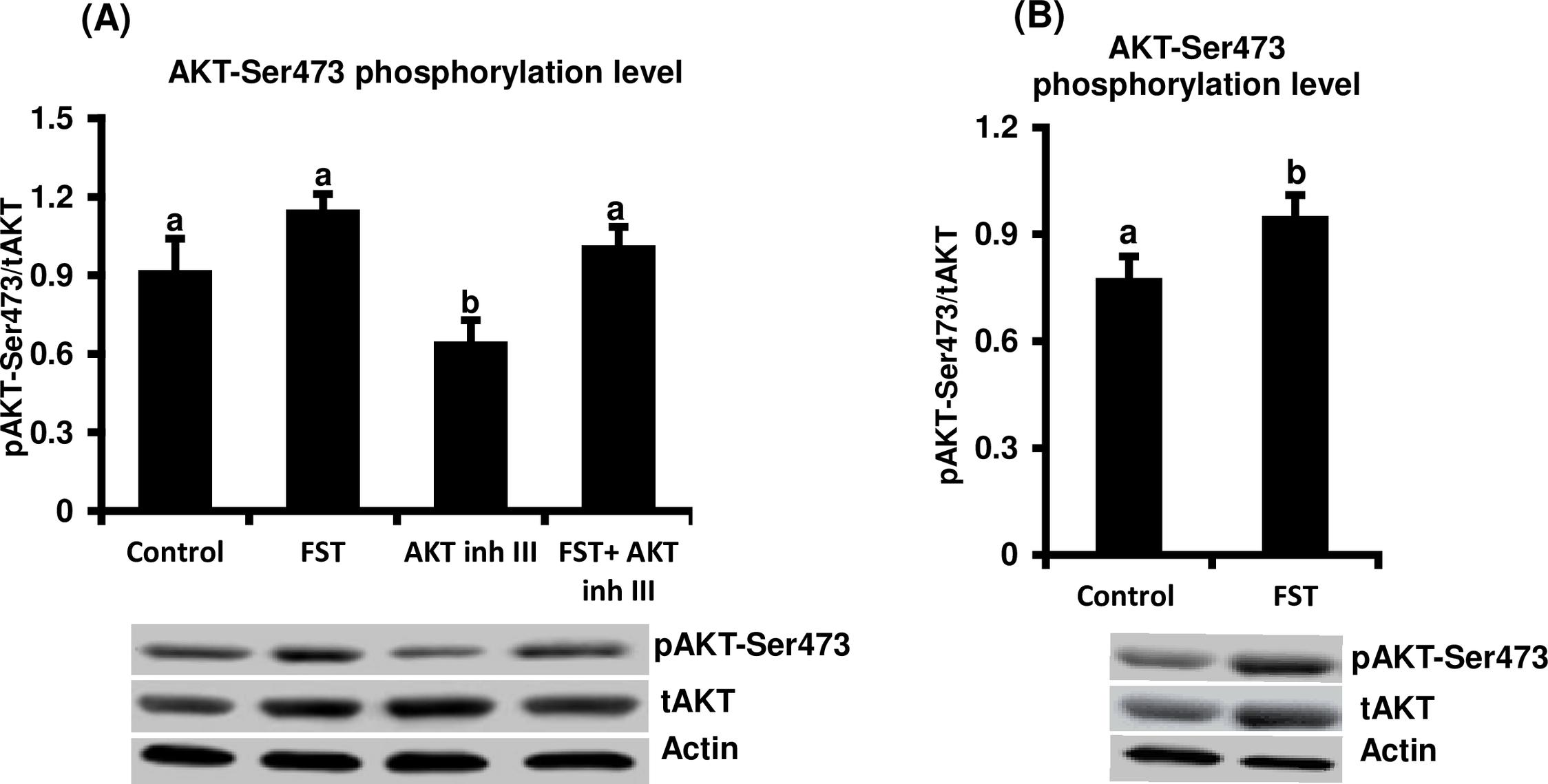

Supplement: Supplementary file 2 — Effect of follistatin treatment on AKT-Ser473 phosphorylation in early bovine embryos. Presumptive zygotes were cultured with 0 or 10 ng/ml recombinant human follistatin in the presence or absence of AKT inhibitor III (75 μM) for 10 h (n = 5 replicates, n = 20 zygotes/group) (a), or Presumptive zygotes were cultured in the presence or absence of 10 ng/ml follistatin for 24 h (n = 5 replicates, n = 20 zygotes/group) (b). Samples were subjected to Western blot analysis for pAKT-Ser473, tAKT and Actin. Expression levels were normalized to the abundance of an endogenous control actin. Phosphorylation level was expressed as pAKT/tAKT. Data are expressed as mean ± standard error. Values with different superscripts among treatments indicate significant differences (P < 0.05). Representative Western blots are shown. (TIFF 542 kb) [file 12958_2017_318_MOESM2_ESM.tif]
